# Impact of the Wood Species Used on the Chemical Composition, Color and Sensory Characteristics of Wine

**DOI:** 10.3390/foods14122088

**Published:** 2025-06-13

**Authors:** Ana María Martínez-Gil, Maria del Alamo-Sanza, María Asensio-Cuadrado, Rubén del Barrio-Galán, Ignacio Nevares

**Affiliations:** 1 Department of Analytical Chemistry, UVaMOX-Higher Technology Collage of Agricultural Engineering, Universidad de Valladolid, 34001 Palencia, Spain; anamaria.martinez.gil@uva.es (A.M.M.-G.); maria.asensio.cuadrado@uva.es (M.A.-C.); ruben.barrio@uva.es (R.d.B.-G.); 2 Department of Agricultural and Forestry Engineering, UVaMOX-Higher Technology Collage of Agricultural, Universidad de Valladolid, 34001 Palencia, Spain

**Keywords:** white wine, *Quercus candicans*, *Quercus humboldtti*, *Nothofagus pumilio*, *Prunus avium*, *Robinia speudoacacia*, *Acacia dealbata*

## Abstract

In recent decades, the use of wood pieces has been promoted as a viable alternative to barrels to improve the quality of white wines. However, most available studies have focused on red wines. Given that white and red wines present significant oenological differences that affect their development and final characteristics, it is necessary to expand research specifically to the case of white wines. For this reason, this study evaluates the impact of using pieces of traditional oak wood (*Quercus petraea* (two origins: French and Romanian) and *Quercus alba*), other oaks (*Quercus humboldtti* and *Quercus candicans*) and other genera (*Robinia pseudoacacia*, *Acacia dealbata*, *Prunus avium* and *Nothofagus pumilio*) on the quality of white wine during the short period of contact with the wood. The results show that aging with the different woods has little effect on the oenological parameters of the wine; however, it does lead to a change in the phenolic composition and in the final chromatic characteristics of the white wines. From a sensory point of view, the wines showed different sensory profiles depending on the type of wood used. In general, the tasting panel preferred the white wine aged with French *Quercus petraea* wood pieces, followed by the wine aged with *Quercus humboldtti* wood pieces and the wine aged with *Robinia speudoacacia* wood pieces. This research improves our understanding of the potential impact of using pieces of different woods in white wines, describing the potential interest of some that have not been studied before, such as *Quercus humboldtti*.

## 1. Introduction

The use of wood during the fermentation and aging processes in wine production causes significant changes in its chemical composition and sensory properties, modifying its color, aroma, flavor, and the stability of key wine attributes [1,2]. Oak has been one of the main woods used for wines, specifically the European species *Quercus petraea* and *Quercus robur*, and from the American continent, *Quercus alba* [2,3]. In the past, due to the lack of oak, many woods were used; however, today only oak and chestnut are recommended by the International Organization of Vine and Wine (OIV) for barrels [4] and only the genus *Quercus* for the use of wood chips [5]. However, in the last two decades, the use and/or study of alternative oaks (other species) or other woods (other genera) is being considered as a solution to the search for new sources of wood for cooperage, since the current high demand for oak wood may have a negative ecological impact on oak forests, with the replanting of trees not being guaranteed [2,6]. Furthermore, this growing demand for oak has caused a notable increase in costs for cooperages due to the limited availability of material [2,6].

The search for new wood species is not only a response to the limited availability of oak, but also to the growing saturation of the wine market. In this context, winemakers are seeking to innovate by using alternative woods that allow them to produce wines with distinct profiles. This approach aims to identify new wood varieties with oenological potential for use in winemaking. For all these reasons, the cooperage industry is obliged to offer the widest variety of wood products for use in the production of wines and other beverages. Thus, in recent years, the possibility of using other non-traditional Quercus (authorized as *Quercus*) such as *Q. faginea* Lam., *Q. pyrenaica* Willd, *Q. farnetto* Ten., *Q. oocarpa* Liebm., *Q. pubescens* Mill. and *Q. humboldtii* Bonpl, including other wood species (not authorized by the European Union or the OIV) such as *Robinia pseudoacacia* L., *Prunus avium* L. and *Prunus cereasus* L., *Fraxinus excelsior* L., and *Fraxinus americana* L. and *Nothofagus Pumilio* have been the subject of studies, especially in red wines, while for white wines there is very limited knowledge [2,3,6,7,8,9,10].

The white wine market has been dominated for many years by young varietal wines, designed to be consumed shortly after bottling in order to preserve their freshness and fruity character. In these types of wines, the decrease in varietal aromatic compounds—such as terpenes, methoxypyrazines, or volatile thiols—is commonly associated with a loss of sensory quality [7,8]. Furthermore, in the case of white wines, oxidation causes a sensory decrease in the level of positive attributes, together with the appearance of unpleasant flavors, such as “honey” or “cooked vegetables” and browning of the wine [8,11]. Occasionally, barrel fermentation or aging on lees has been used in the production of white wines [12,13]. However, an increasing number of studies have focused on the contact of white wine with wood, both during alcoholic fermentation and the subsequent barrel aging. Aging in barrels allows wine micro-oxygenation thanks to the entry of air mainly through the wood and through the joints between the staves, which depends on many factors such as the type of wood, its anatomy, toasting, uses, environmental conditions of the cellar, etc. [14], and which defines the characteristics of aged wines. Since the year 2000, much work on the use of wood chips in winemaking has been published, especially on the impact they have on the chemical composition and sensory characteristics of red wines. Nowadays, the use of wood chips to improve the quality of white wines has become increasingly popular as a good alternative to the use of barrels for the aging of white wines [6,7,8,15,16,17,18,19,20,21,22]. The studies on white wines has focused on Chardonnay wines [13,16,21,22,23], although there are also studies on Verdejo wines, Sauvignon blanc, Malvazija istarska, Encruzado, Listan… [15,17,20,22,24,25]. The woods studied for the manufacture of barrels for white wines have been *Robinia pseudoacacia* [26,27,28] and *Robinia speudoacacia*, *Quercus pyrenaica*, *Castanea sativa* and *Prunus avium* for alternatives [6,7,8,15,23,28].

The aging of wines with *Nothofagus pumilio* and *Quercus humboldtii* wood has been little studied, with studies having been carried out on red wine [9,29]. Furthermore, nothing has been found in the literature on the aging, neither in red nor white wines, with *Acacia dealbata* or *Quercus candicans* wood. These non-traditional woods (alternative woods) differ significantly in their chemical composition as well as in their physical and mechanical properties—such as porosity, grain size, and flexibility—which together influence the transfer of compounds between wood and wine [3,6,7,8,10,15,29].

This work is presented as an opportunity to evaluate the impact of traditional oak wood (*Quercus petraea* (two origins) and *Quercus alba*), other oaks (*Quercus humboldtti* and *Quercus candicans*) and other genera (*Robinia speudoacacia*, *Acacia dealbata*, *Prunus avium* and *Nothofagus pumilio*) on the quality of white wine during a short period of contact with the wood. Therefore, this study addresses a notable gap in the current literature by systematically evaluating the effects of several new or understudied wood species on white wine quality parameters, thereby expanding knowledge beyond the traditional focus on red wines and conventional oak species.

## 2. Materials and Methods

### 2.1. Woods

Different alternative wood pieces were used: *Robina pseudoacacia* from Romania (RP), *Acacia dealbata* from Chile (AD), *Prunus avium* from Romania (PA), *Nothofagus pumilio* from Chile (NP), *Quercus candicans* from Mexico (QC), *Quercus humboldtti* from Colombia (QH). In addition, wood pieces of traditional oaks have been used—*Quercus petraea* from Romania (QPR), *Quercus petraea* from France (QPF) and *Quercus alba* from Kentucky (QA). The nine woods were supplied by the company TN Coopers of the Metropolitan Region (Chile). All of them were obtained using the sawing technique, except for the *Quercus petraea* species, which was obtained by splitting. All the alternative woods were medium-coarse-grained, while the traditional woods were medium-fine- (American) or fine- (French and Romanian) grained. The cooperage dried these woods naturally, exposing them to the sun, rain and open air. During the summer months they were watered for long enough to bring their moisture content down to between 11 and 13%. This took 6 months for AC, PA, QC woods, 8 months for AD, 12 months for NP, 26 months for QA, 25 months for QH and 30 months for QPR and QPF. After seasoning, the staves were cut to 16 × 7.8 × 2 cm and toasted with the same toasting method using an industrial convection oven. The wood was stored in conditions of suitable relative humidity and temperature until use (barrel room, 72 ± 3% 19 ± 1 °C). A few days before use, they were cut to the dimensions required for testing, 1.5 × 7 × 1.6 cm.

### 2.2. White Wine and Experimental Conditions

A young white wine of the Verdejo variety, vintage 2023, from a vineyard in the Rueda Designation of Origin, was used. The young wine was produced in a commercial winery. The oenological characteristics of white wine are shown in Table 1.

The white wine essay for each wood (600 mL glass containers in triplicate and one glass container of 3000 mL) were in contact with the different wood piece species for 20 days at cellar temperature (16.5 ± 0.5 °C) and stirred manually once a week for 5 min. The white wines in contact with the different woods were named as follows: **RPw** (wine+*Robina pseudoacacia*) **ADw** (wine+*Acacia dealbata*), **PAw** (wine+*Prunus avium*), **NPw** (wine+*Nothofagus pumilio*), **QCw** (wine+*Quercus candicans*), **QHw** (wine+*Quercus humboldtti*), **QPRw** (wine+Romanian *Quercus petraea*), **QPFw**, (wine+french *Quercus petraea*) and **QAw** (wine+*Quercus alba*). The wood-to-wine ratio of 80.3 cm^2^/L that was established is equivalent to an exposure surface area of 2/3 of the usual surface area of a Bordeaux barrel.

To achieve this, a piece of previously prepared wood (1.5 × 7 × 1.6 cm) was added to every 600 mL of wine, while for the 3000 mL tanks it was necessary to add 5 pieces with the same dimensions to achieve a ratio of 80.3 cm^2^/L. For the experiment with 9 woods, 6 alternative woods and 3 traditional woods, 27 glass containers of 600 mL (9 woods × 3 repetitions) and 9 glass containers of 3000 mL (9 woods × 1 repetition) were needed. In addition, a trial without the addition of wood, called a control sample, was carried out simultaneously, occupying 3 glass containers of 600 mL and 1 glass container of 3000 mL during the same time and under the same conditions. This wine is called **CTw** (wine without wood).

After 20 days, the wood was removed and all wines, both the three 600 mL replicates and the single 3000 mL sample, were chemically analyzed separately. For sensory analysis, all replicates from each wood species were combined to create a homogeneous sample, which was evaluated one month after bottling.

### 2.3. Chemical Analysis

The wine studied was analyzed initially (Table 1) and after 20 days of contact with the different woods. The general white wine physical–chemical characterization, pH, total acidity (g/L tartaric acid), volatile acidity (g/L acetic acid), free sulfur dioxide (F-SO_2_, mg/L), total sulfur dioxide (T-SO_2_, mg/L), and reducing sugars (g/L) were measured following the methods indicated by International Organization of Vine and Wine [30].

All trial samples with each of the 9 woods were analyzed in duplicate (n = 8, 4 wines and 2 analyses).

#### 2.3.1. Spectral Analysis

The ultraviolet and visible spectra of the wines were measured before and after contact with the wood. Quartz cuvettes with an optical path length of 1 cm were used in a PerkinElmer Lambda 25 ultraviolet-visible (UV-visible) spectrophotometer (Waltham, MA, USA). Pure water was used for the reference scan. Absorbance spectra in the range of 350 to 780 nm, with measurements at 5 nm intervals, allowed the analysis of the color at 420 nm and the intensity of the color corresponding to this wavelength according to the method described by Glories et al. [31]. The CIELab parameters were calculated using the OIV standard method OIV-MA-AS2-11 and included: L* (lightness, from black to white), a* (color from red to green), b* (color from blue to yellow), C* (chroma or saturation) and H* (hue angle) [30]. The browning of the wines after 20 days of aging was analyzed following the method by De Coninck et al. [32], subjecting the wines to a temperature of 50 °C in an oven.

#### 2.3.2. Phenolic Analysis

Total phenolic compounds (TPs) were determined following the Folin and Ciocalteu method [33], and low polymerized phenols (LPPs) using the Masquelier and Michaud method [34]. Highly polymerized phenols (HPPs) were calculated as the difference between TPs and LPPs. Tartaric esters and flavanols were determined according to Mazza et al., [35]. Finally, total tannins (TAN, as g/L of cyanidin chloride) were analyzed using the Riberéau-Gayón and Stonstreet [36] method. All of them were measured using quartz cells with a path-length of 1 cm and a PerkinElmer Lambda 25 UV-visible spectrophotometer (Waltham, MA, USA).

### 2.4. Sensory Analysis

The analysis was carried out in the tasting room located on the Yutera Campus of the Higher Technical School of Agricultural Engineering of Palencia, a room that complies with the UNE-EN ISO 8589:2010/A1:2014 [37] standard. Two types of sensory analyses were conducted: (1) a hedonic preference test and (2) a descriptive analysis to characterize the visual, olfactory, and gustatory profiles. The ordering analysis consisted of ordering the different wines by smell and taste, evaluating from highest to lowest preference and finally choosing the best and worst wine in terms of overall preference. The hedonic preference test involved 28 consumers (16 men and 12 women, aged 24–66) who ranked the wines by aroma and taste from most to least preferred, ultimately selecting the most and least preferred wines overall. The descriptive sensory analysis was performed by 17 expert tasters (9 men and 8 women, aged 36–66), who evaluated the wines’ sensory attributes in detail.

The two types of analysis were carried out on different days. Prior to tasting, the samples (i.e., the four tanks made with the same type of wood) were homogenized and bottled in 750 mL dark green bottles. After one month in the bottle, the bottles were opened ten minutes before tasting. The samples were served at a temperature of 16–18 °C. In each session, the tasters evaluated 10 wines (6 from the alternative woods, 3 from the traditional woods and 1 without contact with wood). The glasses were identified with three-digit codes, chosen at random and different from one sample to another. The glasses were filled with about 75 mL of wine. The wines were preferentially ranked, giving the favorite a score of 10 points and the least favorite a score of 1 point for both smell and taste, and then each taster indicated which wine was the best and which was the worst according to their overall assessment. In the scaled tasting, a score of 0 to 5 was given to each attribute, where 0 = not perceptible; 1 = beginning to be perceptible; 2 = weak; 3 = moderate; 4 = strong; 5 = very strong. In the visual phase, color intensity, yellow, green, and golden were evaluated; in the olfactory phase, olfactory intensity, frankness, fruity, floral, vegetable, wood, toasted, vanilla, spicy, dried fruits, and complexity were the attributes evaluated and in the gustatory phase, acidity, bitterness, astringency, body, persistence, and balance were studied.

### 2.5. Statistical Analysis

The statistical elaboration of the data was performed using IBM SPSS Statistics 29.0.2.0. The statistical analysis of each of the parameters evaluated (chemical and sensory) was carried out in order to compare the wines aged in contact with the different types of wood and also with the control wine (without wood). Differences between means were compared using the minimum significant difference test (Tukey) at the 0.05 probability level. The results of the analysis of variance (ANOVA) are presented using different letters to indicate significant differences between the different wines after 20 days.

## 3. Results and Discussion

### 3.1. General Chemical Characteristics

The general chemical attributes in Verdejo wines after 20 days without or with different types of woods are presented in Table 2. Classical oenological parameters were studied because some are directly related to wine quality and stability such as volatile acidity, ethanol, pH, total acidity, and SO_2_ levels and because they are closely related to the extractive capacity of wines such as ethanol content and pH [38]. Contact with pieces of different woods did not affect the alcoholic content and had minimal effect on the reducing sugars. The differences found in sugars between the RPw aged wine compared to the **NPw**, **QCw** and **QAw** wines were not of oenological importance, as the maximum difference was 0.10 g/L, with these being considered dry wines. With regard to pH, an increase was observed in all the wines after 20 days of aging (Table 1 and Table 2), being higher in the wines in contact with wood compared to the wine without wood contact, although without significant differences with the **RPw**, **QHw**, **QPRw** and **QPF** wines. The wine that increased the most was **QCw**, with an increase of 0.16, followed by **NPw**, which is similar to the wine with traditional wood **QAw**. The wines that showed the highest pH (**NPw**, **QCw** and **QAw**) were those that showed a drop in total acidity of 0.20 g/L compared to the control wine, which remained stable over time (Table 2). All the other wines in contact with the wood had an increase in total acidity of 0.20 g/L in the **RPw** wines to 0.40 g/L in the **QHw** and **QPEw** wines. What usually happens during the aging of wines is a decrease in total acidity due to the precipitation of tartaric acid [39]; however, with wood there can be a transfer of acids which can lead to an increase in acidity [40]. The volatile acidity of the wines showed an increase in the case of wines in contact with the different woods, except in the case of wine in contact with *Acacia dealbata* (Table 1 and Table 2). Despite the increase in most of the wines, none of them exceeded the permitted values or the content that is normally detected in sensory tasting. The rest of the wines in contact with the alternative woods showed an increase in volatile acidity, in line with the wines in contact with traditional woods, except for the **RPw** wine which was higher (between 0.05 and 0.10 g/L more than the traditional ones). This increase may be due to acetyl groups from the degradation and depolymerization of hemicellulose during the toasting process being present on the surface of the wood [40,41]. The formation of acetic acid derives from both lignin and hemicelluloses, so the composition of each wood in these compounds will cause different amounts to form during toasting [42]. The F-SO_2_ content decreased considerably compared to the wine without wood contact—while in the wine without wood contact a decrease of 6 mg/L was observed during the 20 days, the wines with wood saw at least twice that decrease (12 mg/L to 17 mg/L). The wines that showed the lowest values were those that were in contact with the alternative woods of *Prunus avium*, *Q. candicans* and *Nothofagus pumilio*, with values similar to when the traditional wood *Q. petraea* was used (**QPRw**). These wines also had the lowest total sulfur content.

### 3.2. Total Phenols

**Figure 1:**
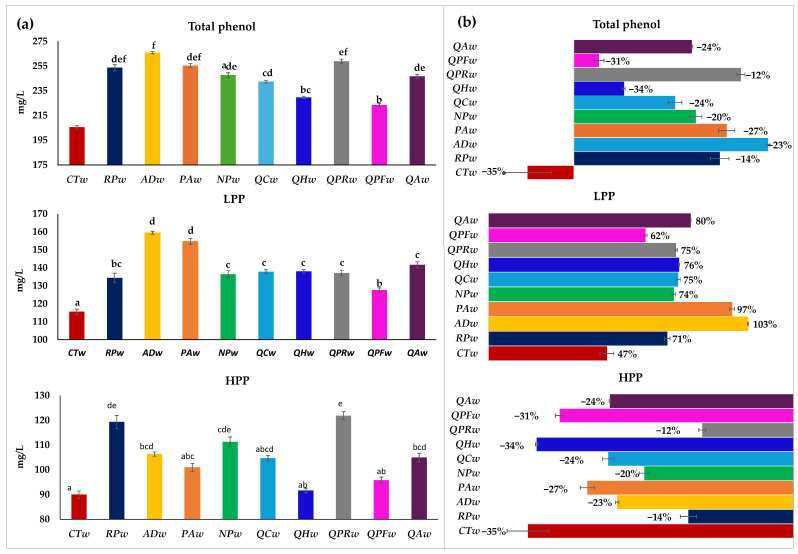
Total polyphenols and degree of polymerization in Verdejo wines aged after 20 days without (control) or with different types of woods. Part (**a**) shows the total content of total phenol, low polymerized phenols (LPP) and highly polymerized phenols (HPP) in the different wines after 20 days; part (**b**) shows the behavior they have had during this period with respect to the initial wine in percentage. Different letters in the same row indicate statistically significant differences between all wines (*p* < 0.05). Wines: **CTw** = control (without wood); **RPw** = *Robina speudoacacia*; **ADw** = *Acacia dealbata*; **PAw** = *Prunus avium*; **NPw** = *Nothofagus pumilio*; **QCw** = *Q. candicans*; **QHw** = *Q. humboldtti*; **QPRw** = *Q. petraea* romanian; **QPFw** = *Q. petraea* French and **QAw** = *Q. alba*).

The results of the total polyphenols and degree of polymerization (low polymerized phenols (LPPs) and high polymerized phenols (HPPs), as mg/L of gallic acid in Verdejo wines obtained after storage for 20 days without or with pieces of different woods, are shown in the left (a) of Figure 1. On the right of the figure (b), their behavior during this period, with respect to the initial wine in percentage, is shown. The total phenol content was higher after 20 days in the wines with contact with wood than without contact, which is in line with previous studies [6,15,28], being higher both in the case of poorly and highly polymerized phenols (Figure 1a). Thus, after this period of 20 days, the control wine (**CTw**) presented the significantly lowest value of total phenols (206 mg/L of total), while the white wines in contact with the different pieces of wood showed a content between 223 and 266 mg/L of gallic acid. Jordao et al. [9] observed that aging red wines with different woods after 30 days in contact also led to an increase in phenolic content compared to wine without wood. Furthermore, in Figure 1b it can be seen that the control wine without contact with wood showed a 5% decrease in total polyphenols after 20 days, while the wines in contact with wood showed increases ranging from 3 to 22%, in the **QPFw** and **ADw** wines, respectively. The increase in the total phenol content in wines aged with alternative woods was of the order found with traditional woods, since the wines aged with alternative woods showed increases ranging from 12.52 to 48.72 mg/L in the **QHw** and **ADw** wines, respectively, while the wines aged with traditional woods increased between 6.29 mg/L and 41. 87 mg/L. Therefore, the alternative woods that probably have a higher content or release more phenolic compounds are ***AD***, ***RP*** and ***PA***, and the one that releases the least is ***QH***. Delia et al. [15] also observed a higher phenolic content in white wines aged with *Robinia pseudoacacia* after 20 days of contact and with *Prunus avium* after 28 days than in wines aged with French and American oak. In the present work, all the treatments to the Verdejo wine resulted in a decrease in highly polymerized phenols of between 35% and 12% (Figure 1b).

The wine without contact with wood showed the greatest loss of HPP (35%), but in line with that observed in the **QHw** and **QPFw** wines (34% and 31%, respectively), so the content of these was not significantly different from that of the control wine (90 mg/L **CTw**, 91.6 mg/L **QHw** and 95.8 mg/L **QPFw**). The wines that had the lowest HPP loss were those that were in contact with **RPw** and **QPRw**, and therefore had the highest HPP, 119.4 and 121.9 mg/L, respectively. The decrease in these HPPs in the control wine was related to the increase in poorly polymerized phenols (47%). However, the wines with wood had at least a 15% increase. This increase is an obvious consequence of the transfer of phenolic molecules from the wood to the wine. In fact, most studies indicate a higher phenolic composition in wines in contact with wood when using pieces of oak or other species of wood, even when the contact time with the wood is short [6,8,15,28]. The total phenol content was significantly higher in the wine in contact with Acacia dealbata (**ADw**) wood, which, despite not having the lowest HPP loss, achieved the highest extraction of phenolic compounds due to having higher LPP. On the other hand, the most stable wines over time are probably the wines in contact with the alternative wood Robinia pseudoacacia (**RPw**) and with the traditional wood Q. petraea (**QPRw**) due to their phenolic content, especially because they better maintain the HPP over time.

### 3.3. Total Flavonols, Tartaric Esters and Condensed Tannins

**Figure 2:**
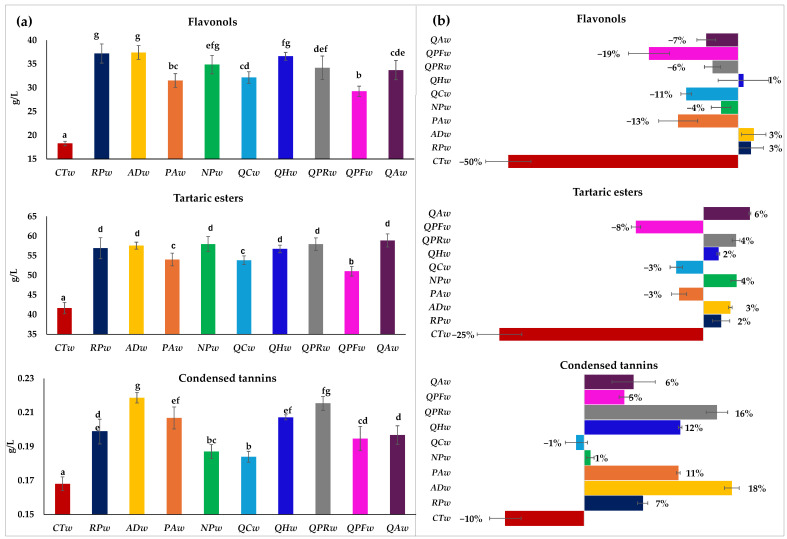
Flavonols, tartaric esters, and condensed tannins in Verdejo wines aged after 20 days without (control) or with different types of woods. Part (**a**) shows the total content of flavonols, tartaric esters and condensed tannins in the different wines after 20 days; part (**b**) shows the behavior they have had during this period with respect to the initial wine in percentage. Different letters in the same row indicate statistically significant differences between all wines (*p* < 0.05)). Wines: **CTw** = control (without wood); **RPw** = *Robina speudoacacia*; **ADw** = *Acacia dealbata*; **PAw** = *Prunus avium*; **NPw** = *Nothofagus pumilio*; **QCw** = *Q. candicans*; **QHw** = *Q. humboldtti*; **QPRw** = *Q. petraea* romanian; **QPFw** = *Q. petraea* French and **QAw** = *Q. alba*).

Figure 2 on the left (a) shows the total content of flavonols, tartaric esters and condensed tannins in the different wines after 20 days without contact with wood (**CTw**) or in contact with different woods, and on the right (b), the behavior they have had during this period with respect to the initial wine in percentage. Flavonols, tartaric esters and condensed tannins all decreased in the control wines (**CTw**), with flavonols being the most affected compounds with a 50% decrease in their initial concentration, from 36 mg/L to 18 mg/L. (Table 1 and Figure 2).

In general, it was also observed that the wines in contact with wood decreased the content of flavonols, but to a lesser extent than in wines without wood (19% to 4%). There are even wines that increase or maintain their flavonol content (**RPw**, **ACw** and **QHw**). These compounds are yellow pigments and therefore contribute to the color of the wines. They are also easily oxidizable compounds, being important antioxidant compounds in white wines since the percentage of these in the total polyphenol content is significant [43]. Woods do not usually contain flavonols in their composition, although they have been detected in *Robinia speudoacacia* wood and also some dihydroflavonol in *Prunus avium* wood [10]. Probably, due to the composition of the *Robinia speudoacacia* wood, a maintenance-increase in the flavonol content was observed in the wine in contact with it (**RPw**) (Figure 2). The wine in contact with Acacia dealbata wood showed the same behavior as the wine with **RPw**, so this wood probably also contains this type of compound in its composition, although it has never been studied after cooperage treatments and the only study found in heartwood is given by total flavonoids without differentiating the type of flavonoids; even so, the content of these is interesting, since it is about 2.5 mg/g of quercetin equivalent [44].

Phenolic acids are mainly esterified with tartaric acid to form tartaric esters and hydroxycinnamic acids in particular are the second most predominant group in must and white wine after flavanols, with the initial Verdejo wine containing 55.7 mg/L. These tartaric acids in the control wine (**CTw**) decreased by 25% to 41.7 mg/L (14 mg/L less), this usually happens because the free acids remain, because they form volatile phenolics or because they oxidize since they are highly oxidizable compounds associated with the browning processes of wine [44]. The same behavior was observed in the **PAw**, **QCw** and **QPFw** wines, but more gradually, since it only decreased by 3% (1.7–1.9 mg/L less) and 8% (4.6 mg/L less) in the **QPF** wine. However, the rest of the wines aged with the other woods showed an increase in tartaric esters of between 2% and 6%, with no differences in the final content between all the wines in which an increase in tartaric esters was observed. This is probably because the wood can release phenolic acids such as ferulic, caffeic and coumaric, which could increase the content and/or improve its stability over time. The results in terms of tartaric acid observed in wines aged in alternative woods are similar to those found in wines aged in traditional woods (Figure 2).

The total tannins in the wine without wood decreased by 10%, while the wines in contact with wood either maintained the tannin content, as was the case with **NPw** and **QCw** wines, or increased it, as was the case with wines in contact with the rest of the woods. Therefore, the lowest contents of this compound were found in wine without wood (**CTw**) followed by **QCw** and **NPw**, although the latter showed no significant differences with QPFw. In a study of red wines where aging with Nothofagus Pumilio cubes and French oak was studied at 30 days, they also observed slightly higher amounts in the wine in contact with French oak but without significant differences with the Nothofagus Pumilio [9]. Furthermore, in this study, the tannins in the control wine decreased over time, while in the wines in contact with wood they increased [9], coinciding with the findings of the present study. The increase can be explained by the transfer of these compounds from the wood to the wine, since wood contains them in its composition. It has been previously described that oak wood usually has few condensed tannins; however, the wood of *Robinia speudioacacia* and *Prunus avium* have a significant content of condensed tannins in addition to tannins not previously described in oak [9,10]. In Figure 2 it can be seen how the condensed tannins in wines in contact with these two woods increase by 7–11%, in general more than traditional woods (5–6%), except for the Romanian oak which is a wood very rich in polyphenols (16%). Correira et al. [6] observed that the content of one of the main precursors of condensed tannins in grapes, catechin, was higher in white wines in contact with *Q. petraea* cubes than with *Robinia speudoacacia*, so it is not only what they yield, but also how they protect the compounds yielded from the wood to those of the wine itself. The wine with the wood that presented the highest content and therefore the greatest increase was the one that was in contact with Acacia dealbata. This wood has not been studied before in the context of wines or the aging of distillates, so there is no analysis after the usual cooperage treatments, but it has been found that heartwood has a significant amount of condensed tannins (4473 mg/g of catechin equivalent) [44]. Condensed tannins play a role in the perception of bitterness, mouthfeel and astringency, especially in red wines since the content in whites is much lower, even so, the tannin content in white wines can contribute to these sensations, especially astringency due to their high acidity [43,45].

### 3.4. Chromatic Characteristics and Browing Potential Index

**Figure 3:**
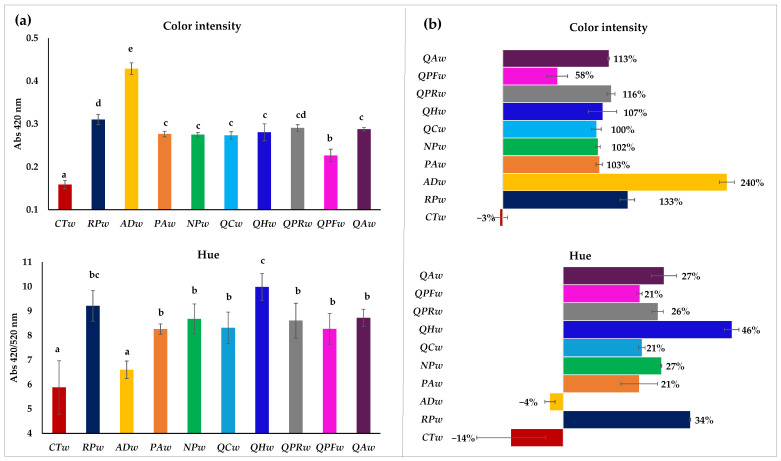
Color intensity and hue of Verdejo wines aged after 20 days without (control) or with different types of woods. Part (**a**) shows the total content of flavonols, tartaric esters and condensed tannins in the different wines after 20 days; part (**b**) shows the behavior they have had during this period, with respect to the initial wine, in percentage. Different letters in the same row indicate statistically significant differences between all wines (*p* < 0.05)). Wines: **CTw** = control (without wood); **RPw** = *Robina speudoacacia*; **ADw** = *Acacia dealbata*; **PAw** = *Prunus avium*; **NPw** = *Nothofagus pumilio*; **QCw** = *Q. candicans*; **QHw** = *Q. humboldtti*; **QPRw** = *Q. petraea* Romanian; **QPFw** = *Q. petraea* French and **QAw** = *Q. alba*).

The color intensity (CI) and hue of the wines after 20 days of contact with the wood are shown on the left of Figure 3a. On the right (b) the behavior they have had during this period with respect to the initial wine in percentage is shown. All the wines showed an increase in CI throughout the aging process with the exception of the wine without contact with wood (**CTw**) which remained constant (0.11 absorbance units). Previous research has reported an increase in the color intensity of white wines when they have been in contact with wood, both in the form of barrels and pieces of wood, due to fact that the compounds that the wood releases, such as furanic aldehydes, react with the compounds that the wine contains, such as flavanols, forming, for example, dimers, trimers and oligomers, which change the color of the wine, especially through the formation of soluble polymeric forms [15,46]. White wine aged in contact with Acacia dealbata wood (**ADw**) presented significantly higher values (0.38 absorbance units), followed by wine aged with *Robinia speudoacacia* wood (**RPw**) (0.26 absorbance units). The aged wines **PAw**, **NPw**, **QCw** and **QHw** showed lower and similar values (between 0.22 and 0.23 absolute units) and no significant differences with the wines aged with traditional woods (**QPRw** and **QAw**), except with **QPFw** which was lower than the wines aged with the rest of the woods (0.18 absolute units). These results are consistent with those observed in Encruzado white wines aged with *Robinia pseudoacacia* and *Prunus avium* cubes and traditional woods, where it was observed that after 20 days of contact with these woods, the wines with *Robinia pseudoacacia* had a higher CI than the *Prunus avium* wines, and that the latter did not show differences with the wines in contact with traditional woods [15]. Therefore, from Figure 3b it can be seen that the majority of the wines increased their absorbance at 420 nm by between 100% and 116%, except for the **QPFw** wine, which increased by 58%, and the wines with acacia, **RPw** and **ADw**, which increased by 133 and 240%, respectively. The **ADw** wine showed the greatest increase in CI and the greatest increase in absorbance at 520 nm (very low values, 0.06, normal in a white wine with no reds). Showing a lighter hue after 20 days with wood, statistically the same as the control wine (**CTw**). However, as usual, the absorbance at 520 nm did not increase either in the rest of the wines with the different woods, or in the **CTw** wine (remaining at 0.02–0.03), therefore the hue of the rest of the wines in contact with wood after 20 days increased (Figure 3b). Consequently, the wines not only changed color due to contact with wood but also their tonality, except in the case of **ADw** which did not change tonality. The increase in the HUE of most of the wines with wood was from 20.6 to 27.3% compared to the initial wine; however, the wine with **RPw** showed a 34.5% increase and the wine with **QHw** a 45.57% increase which made these wines the ones with the highest Hue, although the **RPw** wine showed no other significant differences.

**Figure 4:**
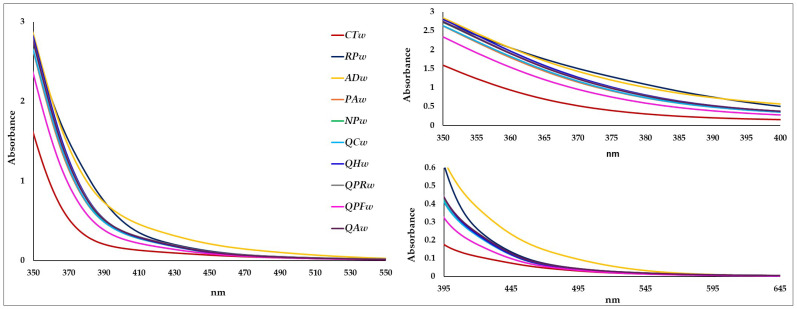
Spectrum of Verdejo wines aged after 20 days without (control) or with different types of woods (wines: **CTw** = control (without wood); **RPw** = *Robina speudoacacia*; **ADw** = *Acacia dealbata*; **PAw** = *Prunus avium*; **NPw** = *Nothofagus pumilio*; **QCw** = *Q. candicans*; **QHw** = *Q. humboldtti*; **QPRw** = *Q. petraea* Romanian; **QPFw** = *Q. petraea* French and **QAw** = *Q. alba*).

The UV-visible spectra of the Verdejo wines, both in contact with different woods and in the wine without wood, were clearly different (Figure 4). There is no significant absorption around 500 nm and less than 0.03–0.0.4 absorbance units (except in the **ADw** wine which was 0.09), as expected for a white wine that does not contain anthocyanins known to have absorbance characteristics at 267–275 and 475–545 nm [47]. The spectra from 335 nm to 470 nm were clearly higher in the wines in contact with wood. From this wavelength onwards, the differences between the control and some wines in contact with wood decreased, with no differences at 495 nm between the control and the wine with **QPFw**, at 505 nm with **NPw** and **QHw**, and at 565 nm with **RPw**, **PAw**, **QCw**, **QPRw** and **QAw** (Figure 4). However, from 335 nm to 595 nm, the spectrum of the **ADw** wine was higher than that of the control wine. Since white wines do not contain significant amounts of flavonoids, the 332 nm peaks can be attributed mainly to hydroxycinnamic acids [47]. In Figure 4 it can be seen that the **PAw**, **ADw**, **NPw** and **QHw** wines presented greater absorbance at 335 nm, which could indicate a higher content of hydroxycinnamic acids [47]. From 345 to 355 nm, it was the **ADw** and **QHw** wines that showed the highest absorbencies (Figure 4). This higher absorbance can be attributed to the content of glycosylated flavonols, since their maximum absorbance is at 350 nm [48]. From 360 nm to 390 nm, it was the **RPw** wines, followed by the **ADw** wine, that showed the highest absorbencies, which could be attributed to a higher content of flavonol aglycones (such as myricetin, quercetin, kaempferol, isorhamnetin, syringetin and laricitrin) which has its maximum absorbance at around 370 nm [48]. However, from the visible range onwards, it was always the **ADw** wine that had the highest absorbance up to 595 nm, where there are no longer any differences.

**Figure 5:**
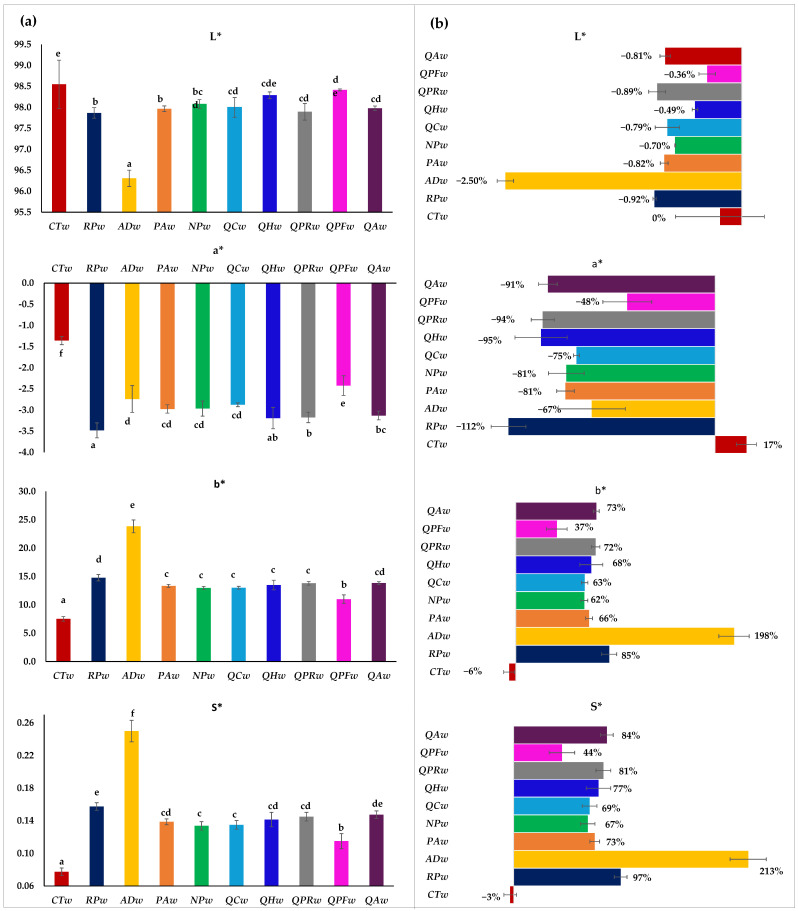
CIELab chromatic characteristics of Verdejo wines aged after 20 days without (control) or with different types of woods. Part (**a**) shows the total content of flavonols, tartaric esters and condensed tannins in the different wines after 20 days; part (**b**) shows the behavior they have had during this period with respect to the initial wine in percentage. Different letters in the same row indicate statistically significant differences between all wines (*p* < 0.05)). Wines: **CTw** = control (without wood); **RPw** = *Robina speudoacacia*; **ADw** = *Acacia dealbata*; **PAw** = *Prunus avium*; **NPw** = *Nothofagus pumilio*; **QCw** = *Q. candicans*; **QHw** = *Q. humboldtti*; **QPRw** = *Q. petraea* Romanian; **QPFw** = *Q. petraea* French and **QAw** = *Q. alba*).

Continuing with the color of the wine, the results obtained with the CIELab method for the chromatic characteristics of the white wines after 20 days are shown in Figure 5a, and on the right (b) their behavior during this period, with respect to the initial wine, is shown in percentage. As for lightness (L*), a decrease was detected over the 20 days, except in the wine without wood (**CTw**), which remained constant. The wines that showed the least decrease in L* were those with the lowest total polyphenol content, i.e., **QHw** and **QPFw**, whose L* did not show significant differences with the **CTw** wine. However, the wine that showed the least decrease in L* was the wine with the highest phenolic content and highest CI, i.e., **ADw**, whose luminosity decreased by 2.5%. As for the a* values (positive related to redness and negative to greenness), they were all negative, which reflects the evident absence of red color in white wines and a tendency towards a greenish color—even more so in this variety, where the color is defined by these shades. The **CTw** wine showed significantly higher values (−0.14 expressed by the CIELab coordinates) as there was even an increase in these coordinates (L*, a*) during the 20-day period. However, the wines in contact with wood caused this coordinate to decrease (between 48% and 112%), especially in wines in contact with Robinia pseudoacacia wood, where the values were significantly different.

Délia et al. [15] also observed a decrease in this CIELab coordinate in white wines in contact with wood. However, Délia et al. found that in wine with *Robinia pseudoacacia* it decreased less than in the wines with *Prunus avium* and traditional oak, exactly the opposite of what was observed in the present study. As for the b* values (coordinate that relates to + for yellows and—for blues), the addition of pieces of wood, regardless of the species used, led to a significant increase in yellow color after 20 days of aging, while the **CTw** wine even shows a decrease in this Cielab coordinate, which confirms the results obtained for color intensity at 420 nm (Figure 3). This b* coordinate showed an increase of almost 200% of the initial wine value when the wine was in contact with Acacia dealbata (Figure 5b), presenting a value of 23.84, followed by the **RPw** wine (14.79), but the latter without significant differences compared to **QAw** (13.85). The rest of the wines showed an increase of approximately 62–72%, so their final value did not show significant differences, except for the **QPFw** wine, which was among the wines with wood that had the lowest CI and also the highest a* and the lowest b* (Figure 5). The increase in this coordinate (b*) of white wines due to them being in contact with wood as opposed to without wood agrees with what has been observed in other studies [15]. It is important to emphasize that the extraction of various phenolic compounds from the wood could also induce an increase in the b* values. Furthermore, this coordinate coincides with previous studies, indicating that Robinia pseudoacacia and Quercus alba increase b* more than Quercus petraea [15]. A similar trend was detected for the saturation values (S*); the control wine presented a saturation of 0.08 compared to 0.25 for the **ADw** wine and all the other wines had an S* of 0.16–0.13 (**RPw** being the upper extreme and **NPw** the lower). Finally, in the wines in contact with wood, the French *Q. petraea* was the one with the lowest saturation value at 0.12.

The results of the browning potential of white wines aged in contact with different woods after 20 days are shown in Table 3. The browning rate in the wines without wood (**CTw**), **NPw**, **QCw** and **QAw** was similar. However, the wines **RPw**, **ADw**, **PAw** and **QPRw** showed significantly higher browning values, followed by **QHw**. Flavonols, such as catechin and epicatechin, caffeic acid and its esters, and gallic acid, are the components of white wine that oxidize most easily [49]. These compounds oxidize to quinones, which are unstable and can undergo further reactions, leading to the formation of brown products. Numerous studies have demonstrated a positive correlation between the flavanol content and the degree of browning of white wines, confirming their involvement in the oxidation process [49]. Furthermore, Barrón et al. [50] observed that a lower flavan-3-ol content resulted in the protection of wines against browning and, consequently, the wines became more stable. In the present study, it is observed that wines with a tendency to browning are those with the highest total polyphenol content (**RPw**, **ADw**, **PAw**, **QPRw** and **QHw**), especially with a higher content of condensed tannins, but, in general, also with a higher content of flavonols and tartaric acids, because these woods are the ones that yield the most or protect these compounds the best, as discussed above. Furthermore, the greater browning of white wines in contact with *Q. petraea*, *Prunus avium* and *Robinia speudoacacia* compared to browning without wood and *Q. alba* has been observed previously, [15], coinciding with what was observed in this study.

### 3.5. Sensory Evaluation

**Figure 6:**
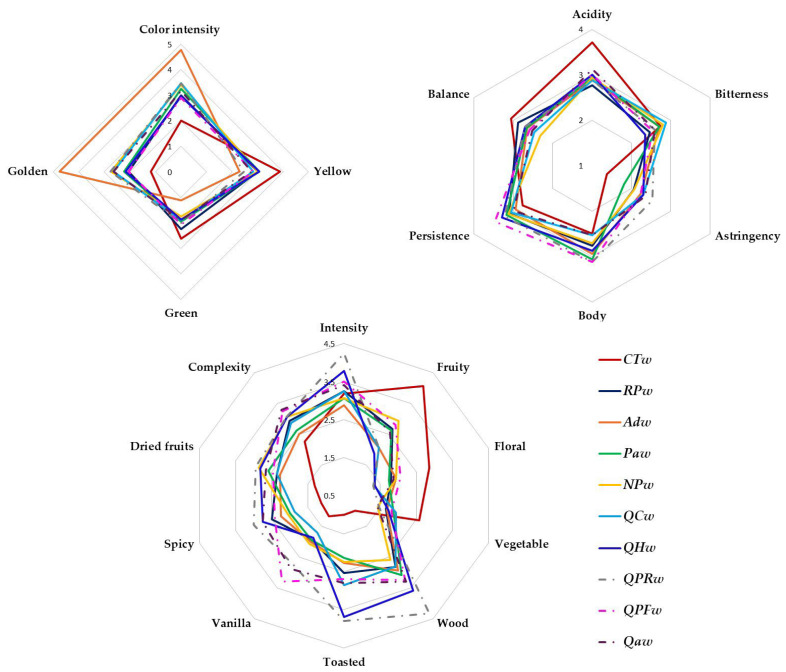
Sensory profile of Verdejo wines aged after 20 days without (control) or with different types of woods (different letters in the same row indicate statistically significant differences between all wines (*p* < 0.05)). Wines: **CTw** = control (without wood); **RPw** = *Robina speudoacacia*; **ADw** = *Acacia dealbata*; **PAw** = *Prunus avium*; **NPw** = *Nothofagus pumilio*; **QCw** = *Q. candicans*; **QHw** = *Q. humboldtti*; **QPRw** = *Q. petraea* Romanian; **QPFw** = *Q. petraea* French and **QAw** = *Q. alba*).

Figure 6 shows the sensory characteristics differentiating the visual, olfactory and taste phases of the different wines after 20 days without contact or with the different woods. The statistical differences in the ANOVA analysis of variance are shown in Table 4. In the visual phase, the tasters observed that the **ADw** wine had the highest color intensity and the control wine the lowest, confirming what the chromatic analyses indicated. However, the tasters defined the wines in contact with **ADw** as having golden hues, but the greenish hue was more noticeable in the **CTw** wine (Figure 6). In the olfactory phase, the wines in contact with wood of **QPRw** followed by **QHw** presented greater olfactory intensity, respectively, with respect to the other wines. This is probably because they were the wines with the most woody and toasted notes, which would indicate that these two woods are capable of transferring from the wood to the wine a higher content of volatile phenols, which are related to these aromas. Therefore, these differences in the chemical composition of the wines could be one of the reasons for the high olfactory intensity of **QPRw** and **QHw** wine and the low olfactory intensity of **ADw** wine. Regarding the concept of “frankness”, i.e., absence of defects, its appreciation was statistically similar in all the wines, which indicates the absence of transfer of wood compounds to the wine that could be perceived as foreign odors and that would lead to a rejection of the wine aged with them. In terms of fruity, floral and vegetal aromas, the control wine was the one that presented the highest score for these attributes compared to the wines with wood, an expected result since the compounds released by the wood cause the primary and secondary compounds to be perceived with less intensity. The wine aged with **NPw** wood best preserved the fruit and floral aromas, indicating that this wood allows the grape’s aromas to be better expressed. Jordao et al., [9] observed that red wines aged with pieces of wood of this species at 30 days also respected the fruit more than other woods. However, the reduction in vegetal notes was observed in all the wines in contact with wood and to the same extent. These aromas can be considered a modification of the olfactory profile of the wines.

The wines that have been in contact with wood showed an increase in the vanilla aromatic note compared to the control, because this compound gives the wood to the wines. The perception of this aromatic note was greater in all the wines in contact with traditional wood than in the wines with alternative wood (Figure 6). Other authors have indicated the null perception of vanilla notes in wines aged with acacia [23]. This could be related to the lower vanilla contents of the alternative woods, which together with the lower or absence of cis- and trans-β-methyl-γ-octalactone content in most of the alternative woods studied, means that vanilla notes are not enhanced [3,23,29]. Both spicy and dried fruit aromas increased in all the wines with wood contact, with the wine aged with the traditional wood **QPRw** scoring highest in these attributes. In the rest of the wines, these attributes were perceived in the wines with alternative wood in the same order as with traditional wood. Wines in contact with wood increased aromatic complexity, with **ADw** and **PAw** showing the least complexity, but in the order of traditional wood **QPFw**.

Regarding the taste phase, aging the wines with wood did not modify the bitter sensations; however, it decreased the sensation of acidity with respect to the **CTw** wine (Figure 6). In addition, higher astringency scores were obtained for all the white wines in contact with the different woods relative to the **CTw** wine, but without statistical differences with those aged with **RPw**, **PAw** and **NPw**. The score for the persistence and body of the wines was increased by the contact of the wine with wood, although, in general, it was only significant with Q. petraea wood. Finally, the most balanced wine was the **CTw** and **QPFw** wine and the least balanced was the wine in contact with **NPw**, all the other samples were in intermediate values to these two samples.

**Figure 7:**
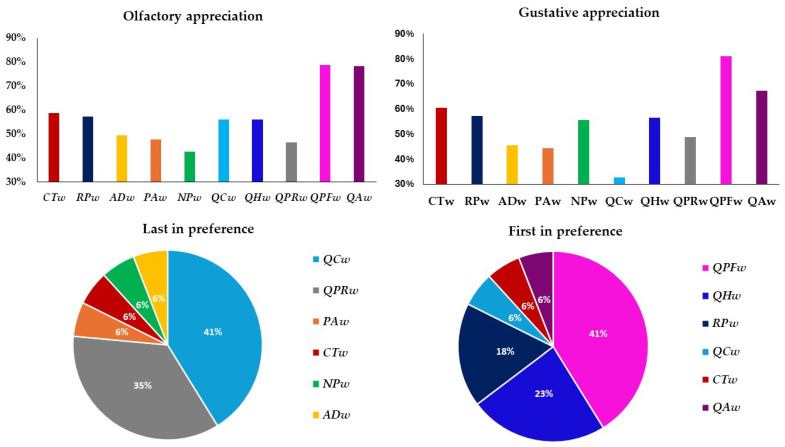
Graph of the percentage score in the olfactory and gustatory phase and preference by the tasters of Verdejo wines aged after 20 days without (control) or with different types of woods. Wines: **CTw** = control (without wood); **RPw** = *Robina speudoacacia*; **ADw** = *Acacia dealbata*; **PAw** = *Prunus avium*; **NPw** = *Nothofagus pumilio*; **QCw** = *Q. candicans*; **QHw** = *Q. humboldtti*; **QPRw** = *Q. petraea* Romanian; **QPFw** = *Q. petraea* French and **QAw** = *Q. alba*.

According to the results, the white wines **QPFw** and **QAw** obtained the best score both olfactorily and gustatorily in the hedonic test (Figure 7). From these results, it could be deduced that the consumer scores more highly the wines that present both olfactorily and gustatorily the attributes to which they are most accustomed, favoring wines in contact with traditional woods. However, when it came to choosing a favorite wine, although the first wine was **QPFw** (chosen by 41% of the tasters), the wine with **QHw** was chosen by 23% of the tasters, and **RPw** by 18% (Figure 7). However, for the first time, it has been observed that another wood, such as that of the *Q. humboldtti* species, could be very interesting in the aging of whites, since, although it did not obtain the best olfactory and gustatory scores, the scores were quite acceptable. At 60%, it was not the worst rated wine by any consumer and, even more so, it was the second favorite (Figure 7). All the other alternative woods (*Acacia delabata*, *Prunus avium*, *Nothofagus pumilio* and *Q. candicans*) presented generally low scores in the olfactory and taste phase, and were not chosen as favorite by any taster—the wines aged with them were selected as the worst, especially **QCw**, which 41% of tasters selected it as the worst wine, indicating that these woods probably lack interest in the aging of white wines (Figure 7).

## 4. Conclusions

This study evaluated the impact of using wood pieces from both traditional and non-traditional species on white wine quality after short contact periods. While the basic oenological parameters were minimally affected, significant differences were found in phenolic composition, chromatic properties, and sensory profiles.

Acacia woods (*Robinia pseudoacacia* and *Acacia dealbata*) significantly increased the total phenolic compound wine content due to higher levels of low-polymerization phenols. Flavonol content showed greater stability in wines in contact with *Robinia pseudoacacia*, *Acacia dealbata*, and *Quercus humboldtii*, suggesting their transfer from wood to wine. Contact with wood allowed the white wine to maintain or even increase its content of tartaric esters, which could contribute to greater stability against oxidation. The highest concentrations of condensed tannins were observed in wines aged with *Acacia dealbata*, *Prunus avium* and *Robinia pseudoacacia*, coinciding with the significant content of condensed tannins reported for these species.

Contact of white wine with *Acacia dealbata* and *Robinia pseudoacacia* led to the greatest increases in CI, attributable to the transfer of phenolic compounds that modified absorbance in the UV-visible spectrum. In general, a decrease in luminosity and in the a* component was observed, together with an increase in the b* component, especially in ***ADw*** wines. These changes highlight the influence of the type of wood used on the chromatic change in white wine.

Sensory analysis revealed distinct profiles among the wines, with those treated with Quercus petraea wood being the most appreciated, followed by those treated with *Quercus humboldtii* and *Robinia pseudoacacia* wood, highlighting the potential of certain non-traditional woods.

This research contributes to a better understanding of how different wood species affect white wine during aging. Among the non-traditional species, *Quercus humboldtii* and *Robinia pseudoacacia* demonstrated notable oenological potential. Future studies should include more detailed analyses of volatile and phenolic compounds to further characterize their influence.

From a practical standpoint, the use of non-traditional woods such as *Robinia pseudoacacia*, *Acacia dealbata*, and *Quercus humboldtii* offers winemakers viable alternatives to traditional oak, enabling the creation of white wines with distinctive chemical and sensory characteristics. These findings may help diversify wine styles and support more sustainable and regionally adapted wood use in enology.

## Figures and Tables

**Table 1:** General physical, chemical, color and phenolic composition of Verdejo wines used in the study.

*Parameters*	
*pH*	3.14 ± 0.06
*Total Acidity (g/L of tartaric acid)*	5.8 ± 0.1
*Volatile Acidity (g/L of acetic acid)*	0.23 ± 0.01
*F-SO_2_ 0(mg/L)*	25 ± 1
*T-SO_2_ (mg/L)*	76 ± 5
*Reducing sugars (g/L)*	0.99 ± 0.06
*Alcoholic content (%)*	12.65 ± 0.24
*Color (Abs 420 nm)*	0.11 ± 0.00
*Luminosity (%)*	98.78 ± 0,11
*Total tannin (g/L)*	0.19 ± 0.00
*Flavonoles (mg/L of quercetin)*	36.21 ± 0.46
*Tartaric sters (mg/L of caffeic acid)*	55.69 ± 1.46
*Total phenolic (Folin) (mg/L of gallic acid)*	217.12 ± 8.48
*LPP (mg/L of gallic acid)*	78.56 ± 3.66
*HMP (mg/L of gallic acid)*	138.56 ± 6.76

F-SO_2_: free sulfur dioxide; T-SO_2_: total sulfur dioxide; low polymerized phenols (LPP); highly polymerized phenols (HPP).

**Table 2:** General chemical in Verdejo wines aged after 20 days without (control) or with different types of woods.

Wine-Wood	pH	Total Acidity (g/L)	Volatile Acidity (g/L)	F-SO_2_ (mg/L)	T-SO_2_ (mg/L)	Reducing Sugars (g/L)	Alcoholic Content (%v/v)
Wine without wood (**CTw**)	3.22 ± 0.02 a	5.8 ± 0.1 b	0.24 ± 0.01 a	19 ± 3 c	79 ± 7 bc	1.11 ± 0.17 ab	12.75 ± 0.19 a
**Alternative woods**							
Wine+*Robina speudoacacia* (**RPw**)	3.23 ± 0.00 ab	6.0 ± 0.1 c	0.40 ± 0.03 f	12 ± 1 b	74 ± 2 abc	1.06 ± 0.02 ab	12.78 ± 0.16 a
Wine+*Acacia dealbata* (**ADw**)	3.24 ± 0.01 b	6.1 ± 0.0 cd	0.26 ± 0.01 a	12 ± 2 b	75 ± 8 abc	1.09 ± 0.04 a	12.79 ± 0.14 a
Wine+*Prunus avium* (**PAw**)	3.24 ± 0.01 b	6.1 ± 0.00 cd	0.34 ± 0.02 de	9 ± 1 a	70 ± 1 a	1.00 ± 0.04 ab	12.68 ± 0.18 a
Wine+*Nothofagus pumilio* (**NPw**)	3.27 ± 0.02 c	5.7 ± 0.1 a	0.29 ± 0.01 b	9 ± 0 a	71 ± 2 ab	1.15 ± 0.08 b	12.83 ± 0.24 a
Wine+*Q. candicans* (**QCw**)	3.30 ± 0.01 d	5.6 ± 0.1 a	0.31 ± 0.02 bc	9 ± 2 a	68 ± 3 a	1.16 ± 0.04 b	12.73 ± 0.16 a
Wine+*Q. humboldtti* (**QHw**)	3.23 ± 0.00 ab	6.2 ± 0.1 d	0.33 ± 0.01 cd	13 ± 3 b	79 ± 10 c	1.13 ± 0.06 ab	12.78 ± 0.12 a
**Traditional woods**							
Wine+*romanian Q. petraea* (**QPRw**)	3.23 ± 0.00 ab	6.2 ± 0.1 d	0.35 ± 0.01 e	8 ± 1 a	71 ± 2 ab	1.13 ± 0.12 ab	12.74 ± 0.16 a
Wine+*french Q. petraea* (**QPF**)	3.23 ± 0.00 ab	6.0 ± 0.1 c	0.31 ± 0.02 bc	13 ± 2 b	82 ± 6 c	1.06 ± 0.04 ab	12.63 ± 0.18 a
Wine+*Q. alba* (**QAw**)	3.27 ± 0.02 c	5.6 ± 0.1 a	0.30 ± 0.02 b	9 ± 1 a	68 ± 3 a	1.19 ± 0.02 b	12.60 ± 0.19 a

The results shown are the average (n = 8) and standard deviation. Different letters in the same column indicate statistically significant differences between all wines (*p* < 0.05). Total acidity as g/L of tartaric acid and volatile acidity as g/L of acetic acid.

**Table 3:** Browning on the Verdejo wines in contact with the different woods compared to the wine without them.

Wines with Different Woods	Browning Rate
Control without wood (**CTw**)	0.0111 ± 0.0021 a
**Alternative woods**	
*Robina speudoacacia* (**RPw**)	0.0197 ± 0.0019 c
*Acacia dealbata* (**ADw**)	0.0199 ± 0.0025 c
*Prunus avium* (**PAw**)	0.0186 ± 0.0012 c
*Nothofagus pumilio* (**NPw**)	0.0111 ± 0.0008 a
*Q. candicans* (**QCw**)	0.0113 ± 0.0015 a
*Q. humboldtti* (**QHw**)	0.0166 ± 0.0008 b
**Traditional woods**	
*Q. petraea european* (**QPEw**)	0.0196 ± 0.0016 c
*Q. petraea french* (**QPFw**)	0.0137 ± 0.0021 ab
*Q. alba* (**QAw**)	0.0105 ± 0.0016 a

Different letters in the same row indicate statistically significant differences between all wines (*p* < 0.05).

**Table 4:** ANOVA of the sensorial parameters studied in Verdejo wines.

	CTw	RPw	ADw	PAw	NPw	QCw	QHw	QPEw	QPFw	QAw
Color intensity	a	bcd	e	bcd	cd	d	bc	d	b	bcd
Yellow	c	b	a	b	b	ab	b	ab	ab	ab
Green	c	bc	a	b	b	b	b	b	bc	b
Golden	a	b	e	bcd	d	cd	bc	d	b	cd
Olfactory Intensity	ab	abc	a	ab	ab	abc	cd	d	bc	abc
Frankness	a	a	a	a	a	a	a	a	a	a
Fruity	d	bc	ab	bc	c	ab	a	ab	c	bc
Floral	d	abc	c	abc	c	ab	ab	a	c	bc
Vegetable	b	a	a	a	a	a	a	a	a	a
Wood	a	bc	bcd	bcd	b	bc	d	e	bcd	cd
Toasted	a	bc	bc	b	bc	c	d	d	bc	bc
Vanilla	a	b	b	b	b	b	b	c	c	c
Spicy	a	bcd	bc	b	b	b	cd	d	bcd	cd
Dried fruits	a	ab	ab	ab	ab	ab	ab	b	ab	ab
Complexity	a	bc	ab	ab	bc	bc	bc	bc	c	c
Acidity	b	a	a	a	a	a	a	a	a	a
Bitterness	a	a	a	a	a	a	a	a	a	a
Astringency	a	ab	b	ab	ab	b	b	b	b	b
Body	a	ab	ab	ab	ab	ab	ab	b	b	ab
Persistence	a	ab	ab	ab	ab	ab	ab	ab	b	ab
Balance	b	ab	ab	ab	a	ab	ab	ab	b	ab

Different letters in the same row indicate statistically significant differences between all wines (*p* < 0.05).

## Data Availability

The original contributions presented in the study are included in the article, further inquiries can be directed to the corresponding author.
